# Nanoparticle STING Agonist Reprograms the Bone Marrow to an Antitumor Phenotype and Protects Against Bone Destruction

**DOI:** 10.1158/2767-9764.CRC-22-0180

**Published:** 2023-02-08

**Authors:** David C. Florian, Natalie E. Bennett, Mateusz Odziomek, Jessalyn J. Baljon, Mohamed Wehbe, Alyssa R. Merkel, Melissa A. Fischer, Michael R. Savona, Julie A. Rhoades, Scott A. Guelcher, John T. Wilson

**Affiliations:** 1 Department of Biomedical Engineering, Vanderbilt University, Nashville, Tennessee.; 2 Center for Bone Biology, Vanderbilt University Medical Center, Nashville, Tennessee.; 3 Department of Veterans Affairs, Tennessee Valley Healthcare System, Nashville, Tennessee.; 4 Program in Cancer Biology, Vanderbilt University School of Medicine, Nashville, Tennessee.; 5 Department of Chemical and Biomolecular Engineering, Vanderbilt University, Tennessee.; 6 Vanderbilt Ingram Cancer Center, Nashville, Tennessee.; 7 Department of Chemical and Biomolecular Engineering, Vanderbilt University, Nashville, Tennessee.

## Abstract

When breast cancer metastasizes to bone, treatment options are limited. Failure to treat bone metastases is thought to be due to therapy-resistant features of the bone marrow microenvironment. Using a murine model of bone metastatic mammary carcinoma, we demonstrate that systemic delivery of polymer nanoparticles loaded with cyclic dinucleotide (CDN) agonists of stimulator of interferon genes (STING) inhibited tumor growth and bone destruction after 7 days of treatment. Each dose of STING-activating nanoparticles trafficked to the bone marrow compartment and was retained within the tumor microenvironment for over 24 hours, enhancing antitumor immunity through proinflammatory cytokine production and early T-cell activation. While acquired resistance mechanisms, including increased levels of immunosuppressive cytokines and the infiltration of regulatory T cells, ultimately limited antitumor efficacy after 2 weeks of treatment, bone protective effects remained. Overall, these studies demonstrate that STING pathway activation, here enabled using a nanomedicine approach to enhance CDN delivery to bone metastatic sites, can reprogram the immune contexture of the bone marrow to an antitumor phenotype that inhibits bone colonization of metastatic breast cancer cells and protects from tumor-mediated bone destruction.

**Significance:**

Bone metastases are difficult to treat due to the inaccessibility of the bone marrow compartment and the immunosuppressive microenvironment that protects resident stem cells. Packaging a STING agonist into a nanoparticle that enables systemic administration and drug accumulation at tumor sites overcomes both barriers to stymie metastatic breast cancer growth.

## Introduction

Solid tumors commonly metastasize to bone. In particular, 70% to 90% of patients with metastatic breast or prostate cancer have skeletal involvement *post mortem* (1, 2). Malignant cells interact with hematopoietic and immune cells in the bone marrow (BM) tumor microenvironment (TME) to develop therapy resistance and deregulate the natural process of bone turnover, leading to pathologic bone fractures, hypercalcemia, and nerve-compression syndromes (3). In the clinic, the difficult sequela of bone metastases can sometimes be controlled, but not cured.

Immunotherapies have achieved great success in treating a variety of cancer types (4–6), but metastases in the BM are often less responsive to therapies that rely on immune-mediated destruction (7, 8). For example, in a humanized mouse model of B-cell lymphoma, the mAb therapy rituximab eliminated tumor cells in the peripheral blood and spleen but not in the BM (9). Furthermore, an analysis of patients suffering from advanced non–small cell lung cancer with bone involvement had a significantly lower response rate and overall survival when treated with nivolumab (anti-PD1) compared with patients without bone metastases (10). These findings appear to contradict the role of the BM as a primary and secondary lymphoid organ (11, 12), site of monocytopoiesis (13), and a reservoir of memory T cells (14). However, the marrow assumes an immunosuppressive milieu through a high concentration of regulatory T cells (Treg) and anti-inflammatory macrophages and myeloid cells, perhaps to protect resident hematopoietic stem cells (HSC) from self-recognition (15–18). This immunosuppressive microenvironment cannot deter the growth of resident tumor cells (17, 19).

Reprogramming immune cells in the BM by treating with immune agonists or preferentially depleting immunosuppressive cells is a promising approach to eliminating tumors in the BM (8, 20, 21). Combination of mifamurtide, an inflammatory activator of macrophages and monocytes, with chemotherapy reduced the case fatality rate of patients with osteosarcoma by one-third in one clinical study (22). Likewise, combination of the cytotoxic and immunosuppressive drug cyclophosphamide with the mAbs cetuximab or trastuzumab doubled the survival time of mice compared with nontreated or single-agent therapy in a humanized mouse model of bone-metastatic breast cancer (23). The low dose of cyclophosphamide preferentially depleted Tregs and increased the FcγR activatory:inhibitory ratios on macrophages, thereby enabling the mAbs to enhance the antitumor immune response in the BM through antibody-dependent cellular cytotoxicity (23, 24). These studies suggest that the metastatic tumors in the BM niche suppress powerful antitumoral activity of resident innate immune cells, and that this suppression may be overcome with appropriate inflammatory activation.

Stimulator of interferon genes (STING) is a cytosolic pattern recognition receptor that induces production of type I IFNs and IFN-stimulated genes upon binding to its ligand, cyclic guanosine monophosphate–adenosine monophosphate (cGAMP; ref. 25). STING pathway activation has been linked to increased tumor immunogenicity and responsiveness to immune checkpoint blockade in several solid tumor types (26). This has prompted significant recent clinical evaluation of STING agonists based on extensive preclinical validation of therapeutic efficacy in multiple murine models of cancer, including pancreatic cancer (27), melanoma (28, 29), mammary carcinomas (28, 30), and colorectal carcinoma (31), among a growing list of other solid tumors. However, despite their potent antitumor effects, STING agonists have only recently been studied for the treatment of bone metastases in murine models (32). While these studies have demonstrated that STING agonists are potent inducers of metastatic tumor regression, such treatments are not curative. Therefore, the objective of this study was to take a closer look at how STING activation may reprogram immune cells within the BM and in turn how the BM, when seeded with metastatic mammary carcinoma, responds over treatment duration.

Cyclic dinucleotide (CDN) STING agonists such as cGAMP are anionic, highly water-soluble molecules, resulting in poor drug-like properties that limit their membrane permeability and access to the cytosol where STING is localized (33, 34). Consequently, most studies evaluating CDN STING agonists have utilized an intratumoral administration route, typically relying on implantable subcutaneous tumor models. This has also limited the evaluation of CDN STING agonists in murine models of metastatic bone disease owing to the technical and practical challenges of intratumoral administration into bone tumors established in mice. However, recent advances in nanotechnology have enabled encapsulation of cGAMP and other CDNs into drug delivery systems that allow for systemic administration of CDNs (34, 35). These include several liposomal formulations as well as a polymeric nanocarrier platform recently described by Wilson and co-workers (36–38), referred to as STING-activating nanoparticles (STING-NP). STING-NPs have been shown to enhance cGAMP activity by multiple orders of magnitude while also improving pharmacokinetic properties of intravenously administered cGAMP, resulting in enhanced STING activation at solid tumor sites with a concomitant inhibition of tumor growth in multiple subcutaneous tumor models (36, 37). This nanomedicine approach therefore affords an opportunity to evaluate the effects of systemically delivered cGAMP on tumor growth in BM (37, 39).

In this study, we assessed STING-NPs as a cancer immunotherapy in a 4T1 mouse model of breast cancer metastasis to the BM. Systemic delivery of STING-NPs inhibited tumor burden at 7 days resulting in reduced bone resorption at 7 and 14 days. *Ex vivo* imaging of fluorescently tagged NPs indicated that STING-NPs were successfully delivered to the BM. In response to STING-NP treatment, the BM exhibited an acute inflammatory cytokine landscape indicative of STING activation at day 3. By day 7, there were higher levels of activated T lymphocytes in treated mice compared with control. However, many of the antitumoral factors declined at day 14. Furthermore, in treated mice, the number of immunosuppressive Tregs in the diseased BM were 3-fold higher in the treated compared with the control at day 14. These findings demonstrate that STING activation transiently reprograms the BM to an antitumor phenotype during an acute phase response. However, for longer treatment regimens, the dose of STING-NPs may need to be increased, the treatment regimen optimized, and/or used in combination with other immunotherapy agents to allow for robust and durable abrogation of bone tumor growth and prevention of tumor-induced bone destruction.

## Materials and Methods

### Materials

Butyl methacrylate, 4-cyano-4-(phenocarbonothioylthio)pentanoate, poly(ethylene glycol) methyl ether (*M*_n_ = 2,000 Da), dicyclohexylcarbodiimide, 4-dimethylaminopyridine, and dichloromethane were purchased from Sigma-Aldrich. 2-(diethylaminoethyl) methacrylate was purchased from TCI Chemicals. 2,2′-azobis(4-methoxy-2,4-dimethylvaleronitrile) was purchased from Wako Chemicals. Pyridyl disulfide ethyl methacrylate was synthesized as described previously (36, 40). 2′3′-cGAMP was synthesized as described previously (36). Alexa Fluor 647 Maleimide was purchased from Thermo Fisher Scientific.

### Cell Culture

4T1-592 cells were received as a gift from Dr. Florent Elefteriou (Baylor College of Medicine, Houston, TX). The 4T1-592 cell line was selected for this study due to its propensity to metastasize and grow in the BM as described previously (41). After 10 passages in cell culture, 4T1-592 cells underwent *in vivo* reselection to ensure consistent syngeneic bone metastases. Harvested cells were identified as 4T1 bone clones through cell culture adhesion and microscopy. Cells were not authenticated any further. *Mycoplasma* testing was conducted every 10 passages or fewer with Lonza Mycoalert *Mycoplasma* Assay Control (Lonza, catalog no. LT07-518). Prior to injection into mice, cells were cultured at 37°C in an atmosphere of 5% CO_2_ and 95% O_2_ in RPMI1640 (Corning) media with 10% FBS (Hyclone Laboratories) and 1% penicillin/streptomycin (Mediatech).

### Synthesis and Characterization of STING-NPs

Poly[(ethylene glycol)-*block*-[(2-diethylaminoethyl methacrylate)-*co*-(butyl methacrylate)-*co*-(pyridyl disulfide ethyl methacrylate)]] (PEG-DBP) was synthesized and characterized as described previously (36). Briefly, PEG-DBP was dissolved in EtOH at 1,250 mg/mL, followed by addition of cGAMP solution stocked at 50 mg/mL in water. Gradual addition of deionized (DI) water during sonication dispersed the gel into stable nanoparticles. STING-NPs were then cross-linked via addition of 0.5 equivalent dithiothreitol (DTT) relative to pyridyl disulfide ethyl methacrylate (PDSMA) groups. Unencapsulated cGAMP was removed via centrifugal filtration through a 3,000 Da MW CO membrane. To measure cGAMP encapsulation efficiency, an aliquot of STING-NP was diluted in acetonitrile for disassembly and then analyzed via high-performance liquid chromatography, as described previously (38). To label the STING-NPs with Alexa Fluor 647, the nanoparticles were reacted with 1 equivalent DTT to reduce the disulfide bond in the PDSMA, and then reacted with 0.1 equivalents (relative to free thiols) maleimide functionalized Alexa Fluor 647 overnight. Any unreacted dye was removed using 10 kDa molecular weight cutoff (MWCO) centrifugal spin filters.

### Mouse Model of Tumor Establishment in Bone

Balb/c female mice (4–6 weeks old, Envigo) were injected with 2.5 × 10^4^ 4T1-592 tumor cells in 10 μL PBS into the left and right tibiae under isoflurane anesthesia as published previously (41). Mice were treated with either 10 μg of cGAMP encapsulated in STING-NPs in 100 μL PBS (*n* = 8) or 100 μL PBS alone (*n* = 8, control) via tail vein injection once every 3 days until sacrifice. For day 3 endpoints, mice were given their injection of PBS or STING NPs 12 hours prior to euthanasia. Fourteen-day cohorts were given an additional injection 12 hours before euthanasia. Bone destruction was monitored weekly by a Faxitron LX-60 Digital Radiography System and mice were euthanized at 7 or 14 days after tumor injection. *A priori* exclusion criteria were set to disqualify animals both for humane and experimental parameters. Tumor seeding was confirmed by radiography and mice without lesions or lost more than 20% of their weight before the study's endpoint would be excluded. One mouse in the untreated cohort was withdrawn prior to the day 7 endpoint due to paraplegia.

### Microcomputed Tomography

At autopsy, the left tibiae were harvested and fixed in 10% formalin (Thermo Fisher Scientific) for 48 hours at 4°C. A high-resolution microcomputed tomography (μCT) 50 system (Scanco Medical) was used to analyze the mouse tibiae bone volume and microarchitecture. Tomographic images were acquired of hindlimbs in 70% ethanol (70 kVp, 12 μm voxel size, 300 ms integration time). μCT images were reconstructed, filtered (σ = 0.2, support = 1.0), and thresholded at 230. Tibiae were contoured starting 10 slices below the growth plate and continued 100 slices in the distal direction using the Scanco software algorithm. Images of individual tibiae were analyzed using the Scanco Medical Imaging software to determine the morphometric parameters.

### Histology and IHC

Fixed tibiae were stored at 4°C in 70% ethanol prior to decalcification in 10% ethylenediaminetetraacetic acid (EDTA) for 7 days at 4°C. Specimens were then embedded in paraffin, sectioned (5 μm) on a microtome, stained with hematoxylin and eosin (H&E), and examined under a microscope. Tumor burden was quantified using ImageJ software and was reported as a percentage of the total free space in the marrow cavity. For osteoclast counts, bone sections were stained for tartrate-resistant acid phosphatase (TRAP) utilizing a substrate incubation step (0.2 mg/mL Naphthol AS-BI, Sigma-Aldrich) followed by a color reaction (25 mg/mL Pararosaniline dye, Sigma-Aldrich) to form a bright red stain in TRAP-positive cells. Sections were then counterstained with hematoxylin, coverslipped, and examined under a microscope and quantified using ImageJ. Osteoclasts were identified as TRAP-positive, multinucleated cells juxtaposed to bone. IHC was carried out on decalcified paraffin-embedded tibial sections by Vanderbilt's Translational Pathology Shared Resource. Positive staining of FOXP3 was quantified by ImageJ.

### Cytokine/Chemokine Quantification

The right tumor-bearing tibiae from mice treated with STING-NPs or PBS (days 3, 7, and 14, *n* = 5) and from non–tumor-bearing mice treated with STING-NPs or PBS (days 3, 7, and 14, *n* = 4) were snap-frozen and stored at −80°C. Blood was also harvested at sacrifice and was centrifuged at 2,000 × *g* for 10 minutes in a refrigerated centrifuge to remove cells and platelets prior to storage at −80°C. On the day of Luminex analysis, the tibiae were thawed and homogenized with a Kinematica Polytron PT 1300 D in CelLytic MT Cell Lysis Reagent. Samples were centrifuged to remove debris, and the protein concentration in the supernatant was quantified through a Pierce BCA Protein Assay Kit. All bone samples were normalized to 1 mg/mL total protein concentration. Bone homogenates and sera were submitted to the Vanderbilt Hormone Assay and Analytical Service core for Luminex analysis. Analytes from the Millipore MILLIPLEX MAP Mouse Cytokine/Chemokine panel were run on samples. The same lysate was also used for a IFNβ LumiKine Xpress ELISA.

### Flow Cytometry: Myeloid Panel

At autopsy, BM from tumor-bearing STING-NP treated and control mice (days 3, 7, and 14, *n* = 5) was retrieved from the diseased right hindlimb by centrifugation (10,000 revolutions per minute (RPM) for 1 minute). The marrow was resuspended in red blood lysis buffer on ice for 5 minutes. Cells were then filtered, centrifuged, counted, and resuspended at a concentration of 1 × 10^8^ cells/mL. CD3^+^ cells were depleted using a Miltenyi Biotec CD3ε MicroBead Kit, LD Columns, and QuadroMACS Separator. The unlabeled cell fraction (i.e., the flowthrough) was counted, and resuspended at a concentration of 1 × 10^7^ cells/mL in PBS containing 0.5% BSA (Sigma-Aldrich). A total of 100 μL of cell suspension for each flow test was transferred into a 96-well plate, blocked with BD Fc Block, and stained with a Bio-Rad Murine Myeloid Cell No Compensation Flow Panel: Pacific Blue-*CD11b* (clone: 5C6, Bio-Rad, catalog no. MCA711PB, RRID:AB_2927543), RPE-Alexa Fluor 750-*CD11c* (clone: N418, Bio-Rad, catalog no. MCA1369P750, RRID:AB_566465), Alexa Fluor 647-*Ly6C* (clone: ER-MP20, Bio-Rad, catalog no. MCA2389A647, RRID:AB_2137341), and FITC-*Ly6G* (clone: 1A8, Bio-Rad, catalog no. MCA6077F, RRID:AB_2927491)*.* Cells were washed twice and then suspended in PBS containing 0.5% BSA and propidium iodide before analysis on a BD LSR II flow cytometer. Fresh BM was run less than 5 hours after mice were sacrificed. Gating scheme is presented in Supplementary Fig. S1.

### Flow Cytometry: Lymphocyte Activation Panel

At autopsy, BM from tumor-bearing STING-NP treated and control mice (days 7 and 14, *n* = 3) was retrieved from the diseased right hindlimb by centrifugation (10,000 RPM for 1 minute). The marrow was resuspended in red blood cell lysis buffer on ice for 5 minutes. Subsequently, 1 × 10^6^ cells/mL in 100 μL of PBS containing 0.5% BSA (Sigma-Aldrich) were stained with BD Fc Block followed by Alexa Fluor 488-*CD45* (clone: 104, BioLegend, catalog no. 109816, RRID:AB_492868)*,* Brilliant Violet 711-*CD3* (clone: 17A2, BioLegend, catalog no. 100241, RRID:AB_2563945), PerCP-Cy5.5-*CD4* (clone: RM4-5, BioLegend, catalog no. 100540, RRID:AB_893326), Brilliant Violet 421-*CD8* (clone: 53-6.7, BioLegend, catalog no. 100753, RRID:AB_2562558), APC-*CD69* (clone: H1.2F3, BioLegend, catalog no. 104514, RRID:AB_492843), and PE-*CD279* (clone: 29F.1A12, BioLegend, catalog no. 135205, RRID:AB_1877232). All surface marker antibodies were purchased from BioLegend. Viability was measured through Ghost Dye Red 780 (Tonbo, catalog no. 13-0865-T500). Gating scheme is presented in Supplementary Fig. S2.

### Flow Cytometry: Treg Panel

At autopsy, BM from tumor-bearing STING-NP–treated and control mice (days 7 and 14, *n* = 4) was retrieved from the diseased right hindlimb by centrifugation (10,000 RPM for 1 minute). The marrow was resuspended in red blood cell lysis buffer on ice for 5 minutes. A Treg Detection Kit (Miltenyi Biotec, catalog no. 130-120-674) was used for the surface staining of CD4 (VioBlue) and CD25 (APC) and the intracellular staining of FoxP3 (PE). Quantified Tregs were CD4^+^ CD25^+^ FoxP3^+^. Viability was measured through Tonbo Ghost Dye Red 780. Gating scheme is presented in Supplementary Fig. S3.

### Biodistribution of NP Load

Balb/c female mice (4–6 weeks old, Envigo) were injected with 2.5 × 10^4^ 4T1-592 tumor cells in 10 μL PBS into the left tibia under isoflurane anesthesia as reported previously (41). The contralateral tibia served as a non-tumor control and received a 10 μL PBS injection. After 6 days of tumor growth, radiographic images (Faxitron LX-60 Digital Radiography System) were taken of the left tibia to verify tumor establishment. On day 7, mice received STING-NPs functionalized with near-IR fluorophore Alexa Fluor 647 in 100 μL of PBS via tail vein injection. Mice were then immediately imaged post-injection on a Pearl Small Animal Imaging System (LI-COR) and subsequently euthanized at 2, 4, and 24 hours after NP injection. Biodistribution was then evaluated by imaging the organs and long bones *ex vivo*. Regions of interest were analyzed using LI-COR Image Studio.

### Statistical Analyses

G*Power (ver. 3.1.9.7; Heinrich-Heine-Universität Düsseldorf, Düsseldorf, Germany) was used to compute the total sample size for tumor burden experiments from *a priori* power analysis (two-tailed test, α = 0.1, and β = 0.2). The effect size was determined to be large (0.9) due to the potent antitumoral effects of STING-NPs seen previously. Results for the power analysis yielded a sample size of 8 for each condition. After euthanasia, all samples were blinded for analyses and unmasked upon completion of the study. All statistical analyses were performed using Prism version 9 (GraphPad Software, Inc.). Values are presented as mean ± SEM and *P* values unless otherwise specified were *, *P* < 0.05; **, *P* < 0.01; ***, *P* < 0.001; and ****, *P* < 0.0001.

### Data Availability

The data generated in this study are available upon request from the corresponding author.

### Ethics Statement

All animal protocols were approved by Vanderbilt University Institutional Animal Care and Use Committee and were conducted according to NIH guidelines for care and use of laboratory animals.

## Results

**FIGURE 1:**
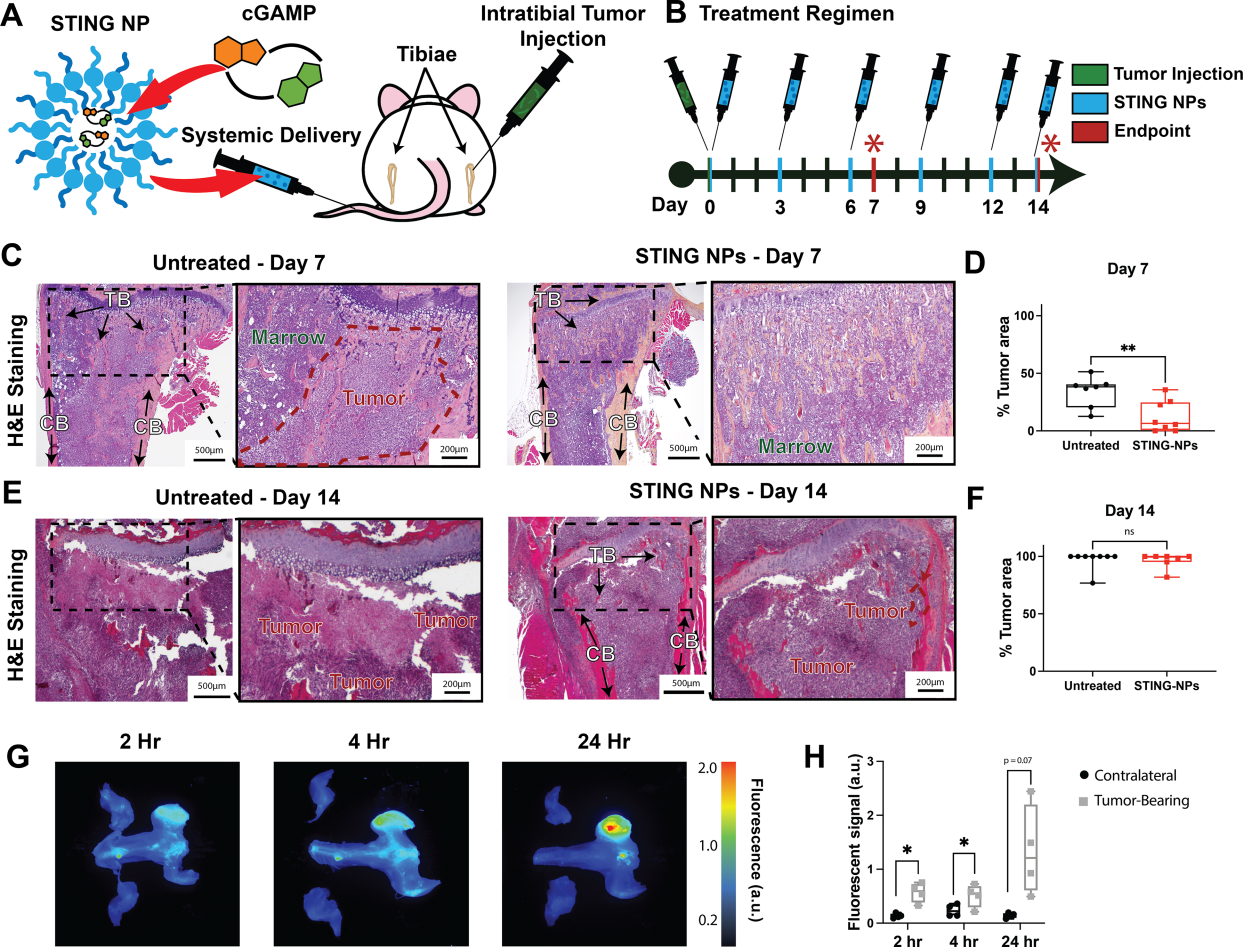
Effects of STING-NP on tumor burden. **A,** Schematic of systemic delivery of STING NPs to murine tumor-bearing tibiae. **B,** Balb/c mice received intratibial injections of 4T1-592 tumor cells on day 0 and were treated with intravenous PBS (control) or 10 μg of STING NPs every 3 days for 7 or 14 days. A final injection was given 12 hours before euthanasia on day 14. H&E-stained sections of tibiae from untreated and STING-NP–treated mice at 7 (**C**) and 14 days (**E**) showing bone (pink/red), BM (blue/purple), and tumor (characterized by dysplasia). Quantification of H&E-stained sections at day 7 (**D**) and day 14 (**F**). At day 14, the tumor filled the marrow space in both control and treated mice. In treated mice, the tumor was contained within the CB, and TB was still present. However, in control mice, the cortical bone was extensively resorbed, allowing the tumor cells to invade adjacent soft tissue. **G,** In a separate cohort (*n* = 4), mice with untreated intratibial tumors were injected with Alexa Fluor 647–labeled NPs at day 7, and the biodistribution of the particles was assessed through *ex vivo* imaging at 2, 4, and 24 hours. Fluorescently labeled particles preferentially accumulated in the marrow. Student *t* test. *, *P* < 0.05; **, *P* < 0.01.

To assess the antitumor effects of cGAMP against solid tumors within the BM, bone-metastatic 4T1-592 mammary carcinoma cells were injected into the tibiae of 6 to 8 weeks old female Balb/c mice and treated with cGAMP encapsulated in STING-NPs or PBS vehicle (untreated) by tail vein injection (Fig. 1A and B). The osteolytic 4T1-592 cell line was chosen due to its propensity to grow rapidly in the BM, causing paraplegia from excessive bone resorption in approximately 2 weeks after intratibial injection (41, 42). A recent pharmacologic study of the systemic effects of STING-NPs demonstrated that a 10 μg dose did not exhibit organ level toxicity when administered every 3 days for 10 days (37). The same treatment regimen was followed in this study but was expanded to 14 days of treatment to match the endpoint of the 4T1-592 model. Tumor-bearing mice were injected with PBS vehicle or STING-NPs on days 0, 3, 6, 9, 12, and 14. It was previously demonstrated that empty nanoparticles lacking cGAMP did not activate STING and, hence, did not exert antitumor effects (37). Mice treated for 14 days tolerated the extended treatment duration well as evidenced by no significant differences in mouse body weight between the control and treatment cohorts (Supplementary Fig. S4).

Tumor burden at days 7 and 14 were assessed by histology and histomorphometry. Histologic sections of the metaphysis on day 7 revealed the presence of tumor (Fig. 1C), which was higher in the untreated group compared with the STING-NP treated (Fig. 1D). By day 14, the medullary cavity in both groups was filled with tumor cells (Fig. 1E), and differences in tumor burden between the two groups were insignificant (Fig. 1F). However, the tumor in the STING-NP treated cohort was contained within the cortical bone (CB), and trabecular bone (TB) was still present, but in control mice the cortical bone was extensively resorbed, allowing the tumor cells to invade adjacent soft tissue (Fig. 1E). Thus, treatment with STING-NPs inhibited growth of 4T1-592 tumor cells at early but not late timepoints.

The low bioavailability of intravenously delivered therapeutics in the bone as a result of lower perfusion compared with soft tissues is an obstacle when treating metastatic tumors (43, 44). The 100 nm diameter of STING-NPs was anticipated to allow these nanoparticles to passively accumulate in tumors through the enhanced permeability and retention effect (36, 45–47). We have previously observed preferential accumulation of untargeted polypropylene sulfide nanoparticles of similar diameter in diseased tibia and hypothesized a similar uptake for STING-NPs (46). To assess the extent of NP uptake by the tumor-bearing tibiae, untreated mice that developed intratibial tumors 7 days prior were intravenously injected with Alexa Fluor 647–labeled NPs. Skeletons were collected at 2, 4, and 24 hours postinjection. Immediately after resection, the hindlimbs were imaged with a LI-COR Pearl Imager, and fluorescent intensity of the tumor-bearing tibiae and contralateral controls were quantified with LI-COR Image Studio (Fig. 1G). Tumor-bearing tibiae exhibited a 4.2-, 2.1-, and 9.5-fold increase in far-red fluorescence at 2, 4, and 24 hours, respectively (Fig. 1H). The high variance in the 24-hour specimens is thought to be a result of the variability in tumor take and tumor burden within the intratibial model, as this is the timepoint when the NPs have been reported to have been mostly cleared from circulation (37). These results demonstrate that labeled STING-NPs preferentially accumulate in tumor sites, even when located in a relatively low perfusion organ.

**FIGURE 2:**
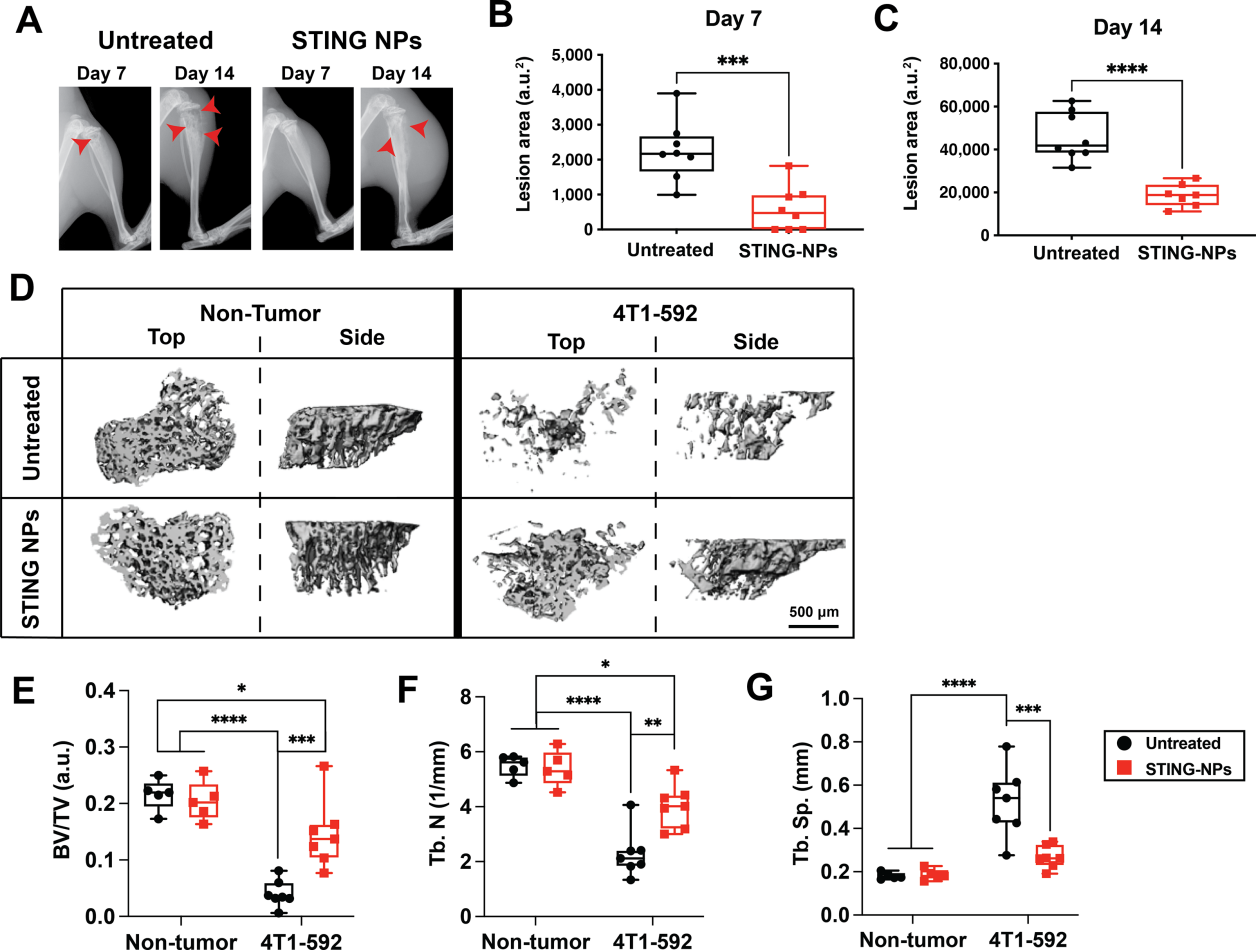
Effects of STING-NP on bone destruction. **A,** Radiographs of untreated and STING-NP–treated tumor-bearing tibiae with osteolytic lesions (red arrows). Quantification of lesion area from radiographs at 7 (**B**) and 14 days (**C**). Student *t* test. ***, *P* < 0.001; ****, *P* < 0.0001. **D,** μCT reconstructions of untreated and STING-NP–treated tibiae from non–tumor-bearing and 4T1-592–bearing mice at 14 days. **E–G,** Bone morphometric parameters BV/TV, Tb.N., and Tb.Sp., calculated from μCT reconstructions at 14 days. Two-way ANOVA with Holm-Šídák multiple comparisons test. *, *P* < 0.05; **, *P* < 0.01; ***, *P* < 0.001; ****, *P* < 0.0001. Error bars: SEM.

Tumor-induced bone resorption can lead to complications such as pathologic fractures and hypercalcemia. Consequently, the effects of STING-NP treatment on bone outcomes were also evaluated on days 7 and 14 (Fig. 2). Osteolytic lesions were observed radiographically in both STING-NP and untreated mice at 7 and 14 days (Fig. 2A). Lesion area was significantly lower in mice treated with STING-NPs at both timepoints (Fig. 2B and C). These radiographic findings were confirmed by μCT analysis. Three-dimensional reconstructions of non–tumor-bearing and tumor-bearing mice treated with STING-NPs or PBS vehicle showed significant bone destruction in the presence of tumor (Fig. 2D). The bone morphometric parameters bone volume/total volume (BV/TV), trabecular number (Tb.N.), and trabecular separation (Tb.Sp.) were quantified using Scanco software. STING-NP treatment did not alter BV/TV (Fig. 2E), Tb.N. (Fig. 2F), or Tb.Sp. (Fig. 2G) in non–tumor-bearing mice. BV/TV and Tb.N. were significantly lower in tumor-bearing compared with non–tumor-bearing mice treated with STING-NPs or PBS vehicle (Fig. 2E and F). While tumor-bearing mice treated with vehicle showed higher Tb.Sp. than non–tumor-bearing mice, treatment of tumor-bearing mice with STING-NPs decreased Tb.Sp. to levels comparable with non–tumor-bearing mice (Fig. 2G). These findings indicate that STING-NPs blocked bone resorption on both days 7 and 14.

In previous studies, Hedgehog signaling inhibitor GANT58 was encapsulated in poly(propylene sulfide) nanoparticles and delivered systemically in a similar bone-tumor model (46, 48). GANT58 inhibited bone resorption in mouse models of bone metastases by reducing expression of the Hedgehog transcription factor Gli2, which regulates PTHrP. However, GANT58 did not inhibit tumor growth, and the bone-protective effect was due to inhibition of osteoclast activity. In contrast, STING-NP treatment reduced tumor burden and the total number of osteoclasts at day 7 (Supplementary Fig. S5A and S5B). However, the number of osteoclasts normalized to the bone that was adjacent to healthy marrow was comparable for both groups (Supplementary Fig. S5B). These observations suggest that reduced bone resorption in response to STING-NP treatment was due to decreased tumor burden on day 7 (Fig. 1D).

**FIGURE 3:**
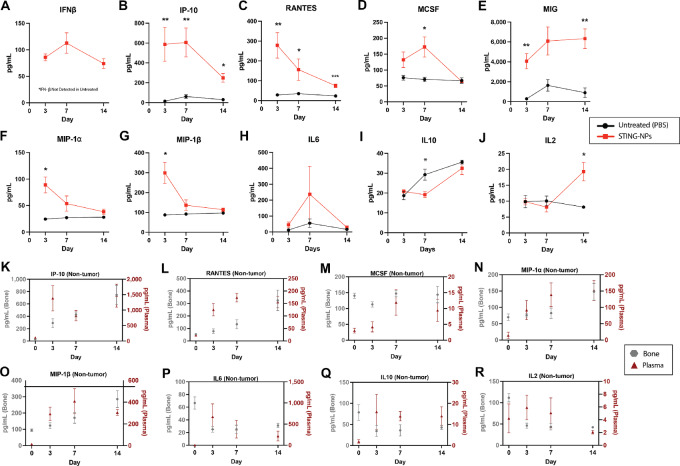
Effects of STING-NP on BM outcomes in tumor-bearing mice assessed by Luminex. **A,** Concentration of IFNβ at 3, 7, and 14 days of STING-NP treatment in 4T1-592 tumor-bearing bone homogenates as measured by LumiKine ELISA (*n* = 5). IFNβ was not detected in untreated samples at the same total protein concentration. **B–J,** Cytokine and chemokines concentrations in tumor-bearing bone homogenates from untreated and STING-NP–treated mice measured by MILLIPLEX MAP Luminex panel (*n* = 5) at 3, 7, and 14 days. Multiple *t* tests with Bonferroni correction. *, *P* < 0.05; **, *P* < 0.01; ***, *P* < 0.001. Error bars: SEM. **K–R,** Non–tumor-bearing mice were treated with the standard STING-NP regimen and the concentration of cytokines in bone homogenates and plasma were measured by MILLIPLEX MAP Luminex panel (*n* = 4) at days 0 (untreated), 3, 7, and 14. All samples (**A–R**) were submitted at a 1 mg/mL total protein concentration for ELISA and Luminex assays. The presence of tumor in the marrow increased the concentration of IP-10 and RANTES by factors of 2 and 3, respectively, at day 3 of STING NP treatment.

STING activation is classically marked by an increase in interferon regulatory factor–driven genes. A MILLIPLEX MAP Mouse Cytokine/Chemokine panel and a standalone IFNβ LumiKine Xpress ELISA were run on lysates from tumor-bearing tibiae resected from mice treated with STING-NPs or PBS on days 3, 7, and 14. STING-NP treatment had higher levels of proinflammatory factors IFNβ, CXCL10 (IP10), and RANTES (CCL5) compared with control at all timepoints (Fig. 3A–C). However, the concentrations of these cytokines were lowest at day 14, suggesting an attenuation of the effects of STING-NPs at longer treatment durations as tumor burden increased. MCSF is higher in treated on days 3 and 7 but not day 14 (Fig. 3D). This decreasing trend was also observed for other chemoattractants important for recruiting immune cells to disrupt tumor growth, specifically MIG (CXCL9), MIP-1ɑ (CCL3), and MIP-1β (CCL4; Fig. 3E–G). IL6, an important factor in bone remodeling and acute inflammatory response, exhibits a trending increase at day 7 but is not significant due to high variability (Fig. 3H). Only IL10 and IL2 exhibited a fold change increase of >1.5 between days 7 and 14 in STING-NP–treated mice (Fig. 3I and J). IL10 is an important mediator of the inflammatory response and negatively regulates STING activation through the STAT3 pathway (49). The increase of IL10 at later timepoints was observed in both treated and untreated cohorts and is likely an immune evasion mechanism deployed by the 4T1 tumor. IL2 exhibits the largest increase from day 7 to day 14 (2.0-fold), and this pleiotropic cytokine has been implicated in both immune tolerance and surveillance. However, a notable role of IL2 is promoting the differentiation, survival, and function of Tregs (50); similarly, IL10 can enhance Treg differentiation and function (51). Taken together, these results indicate that STING-NP treatment induces a potent proinflammatory cytokine response after 3 days of treatment, but after day 7 this response wanes as the BM TME adapts to temper STING-induced inflammation.

Interestingly, this inflammatory decline was not observed in non–tumor-bearing mice that received the same regimen of STING-NPs (Fig. 3K–R). In the non–tumor-bearing experiment, changes in cytokine concentrations in the marrow showed trends similar to the plasma, which increased or remained relatively constant over the 14 days of treatment with the exception of IL6 (Fig. 3P). These data suggest that the tumor plays a role in the resistance of inflammatory signaling at later timepoints. However, without the presence of the tumor mass, fewer NPs will accumulate in the marrow, and these observations could be a result of decreased STING activation in the marrow compartment.

**FIGURE 4:**
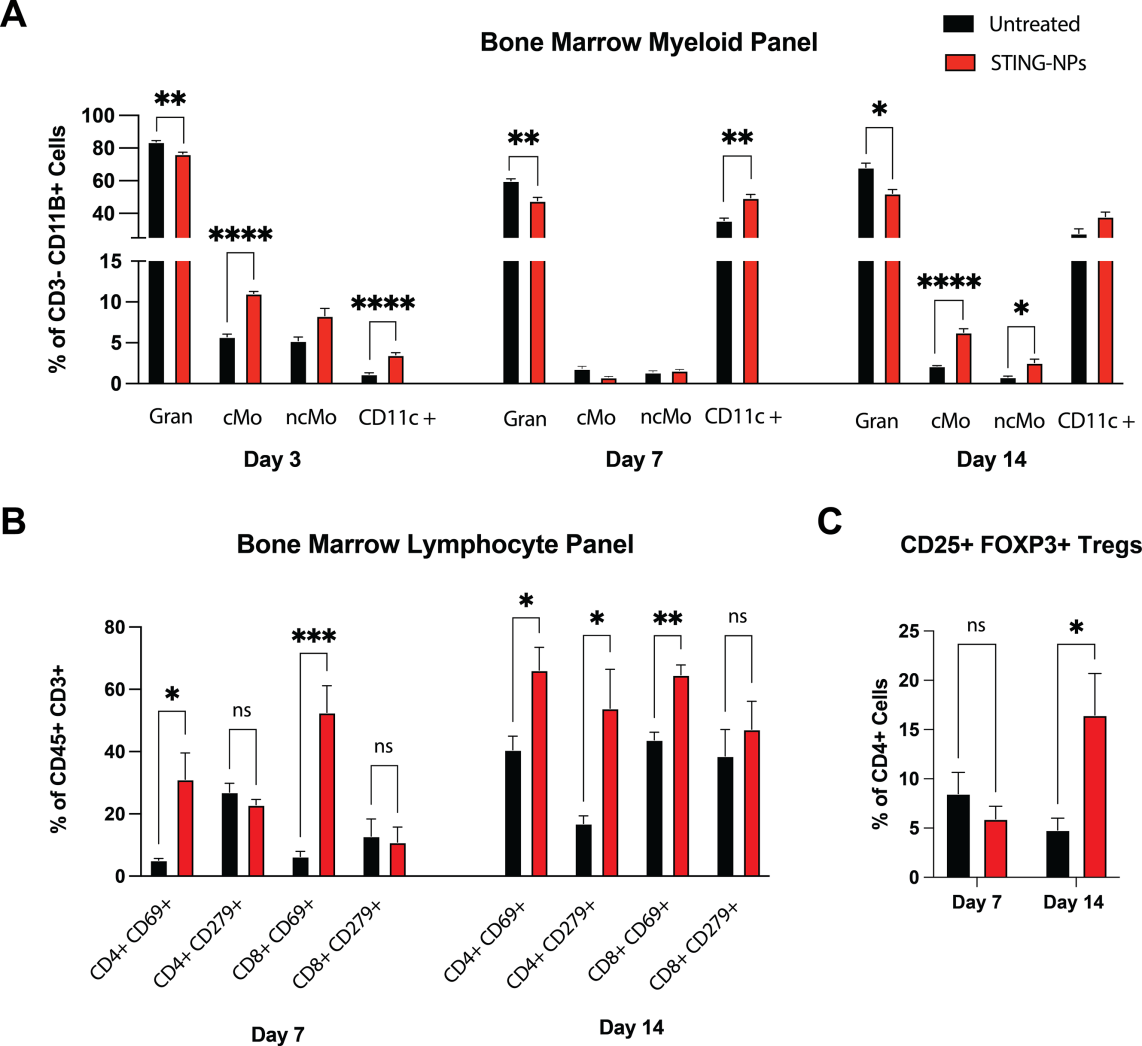
Effects of STING-NPs on BM immune populations. **A,** Flow cytometry analysis of BM aspirates from tumor-bearing hindlimbs of STING-NP–treated and untreated mice at days 3, 7, and 14 (*n* = 6). Granulocytes (Gran: CD11b^+^ CD11c^−^ Ly6G^+^), classical monocytes (cMo: CD11b^+^ Ly6C^hi^ CD11c^−^ Ly6G^−^), nonclassical monocytes (ncMo: CD11b^+^ Ly6C^lo^ CD11c^−^ Ly6G^−^), and CD11c^+^ Cells (CD11c^+^ CD11b^+^ Ly6C^+/−^ Ly6G^+/−^) were quantified as a percentage of CD11B^+^ CD3^−^ cells. **B,** Flow cytometry quantification of T cells in tumor-bearing hindlimbs at days 7 and 14 (*n* = 3). CD69^+^ and CD279^+^ CD8^+^ or CD4^+^ cells are reported as a percentage of total T cells (CD45^+^ CD3^+^). **C,** Tregs (CD4^+^ CD25^+^ FOXP3^+^) cells were measured through flow cytometry at days 7 and 14 (*n* = 4) and reported as a percentage of total CD4^+^ cells. Multiple *t* tests with Bonferroni correction. *, *P* < 0.05; **, *P* < 0.01; ***, *P* < 0.001. Error bars: SEM.

Considering that STING-NPs led to increased expression of proinflammatory and immune cell–recruiting cytokines, we assessed the effects of STING-NPs on the myeloid cell constituency in mouse BM on days 3, 7, and 14 with flow cytometry (Fig. 4A). At all timepoints, the percentage of granulocytes per total myeloid cells was significantly lower in STING-treated BM compared with untreated. A high level of neutrophils, the most abundant granulocyte in the BM, is a poor prognostic factor for patients with primary breast lesions and bone metastases (52, 53). The decreasing granulocyte concentration from days 3 to 7 coincides with a sharp increase in CD11c^+^ cells. Circulating dendritic cells (DC) are readily recruited and retained in the BM during neoplastic insults, which accounts for this growing population in both treated and control groups (54). STING-mediated type I IFN induction further drives the migration and maturation of DCs (55), and as such, we observed a higher concentration of CD11c^+^ cells in the treated cohort at days 3 and 7. DCs are potent targets of cancer vaccines and other immunotherapies for their ability to prime and expand tumor-specific CTLs (56). As the proinflammatory signaling subsides at day 14, the CD11c^+^ population in treated mice is comparable with that of the control group. Further identification of DC subsets is needed to determine the specific cell types that respond to STING agonists.

The concentration of classical monocytes (cMO: CD11b^+^ Ly6C^hi^ CD11c^−^ Ly6G^−^) fluctuated throughout the treatment. From day 3 to day 7, cMO concentration decreased 10-fold and 2.5-fold for the treated and untreated cohorts, respectively. This decrease also corresponds with the increase in CD11c^+^ cells that likely results from either an influx of migratory DCs that dilutes the concentration of cMO or differentiation of cMOs into monocyte-derived DCs or other cells in the monocyte lineage. Regardless, the magnitude of this decrease in cMOs concentrations is proportional with tumor outcomes. The same holds true on day 14, when cMOs increased 6-fold for the STING-treated cohort during a period where the tumor grew to occupy the entire medullary cavity. The TME can recruit and retain monocyte-derived suppressor cells (CD11b^+^ Ly6C^hi^ Ly6G^−^), which is a possible explanation for the suppression of inflammatory factors and poor disease outcome at day 14 in treated mice.

In healthy BM, CD11b^+^ cells sharply decreased in response to initial treatment (Supplementary Fig. S6A and S6B). Despite the low accumulation of STING-NPs in nondiseased BM (Fig. 1G), this tissue is responsive to the systemic effects of STING activation. Therefore, as STING agonists are investigated as therapies for primary tumors, the immunologic response of the BM should be considered. However, the cellular makeup of blood cannot be used as a barometer for STING-mediated effects on myeloid cells in the BM, as less differences were observed in blood than corresponding timepoints in the BM (Supplementary Fig. S7).

The cGAS-STING signaling pathway is a mediator of T-cell activation, trafficking, and cancer cell killing in part through type I IFN production (57). We hypothesized that the elevated IFNβ levels observed in treated BM would correspond to increased T-cell activation (Fig. 3A). Flow cytometry analysis of tumor-bearing BM revealed a significant increase in CD4^+^ and CD8^+^ T-cell activation at day 7 in treated versus control as revealed by CD69 staining (Fig. 4B). Increased adaptive immune response at day 7 corresponds with decreased tumor growth at this timepoint. While this trend continues through day 14, programmed death 1 (PD-1 or CD279), a regulatory immunoreceptor, is significantly higher on CD4^+^ T cells in treated BM compared with untreated. Furthermore, the percentage of CD8^+^ CD279^+^ T cells is 4-fold higher at day 14 compared with day 7 within the STING-NP treated cohort. PD-1 binding with its ligand programmed cell death ligand-1 (PD-L1) inhibits inflammatory cytokine production, T-cell proliferation, and cytotoxic activity (58). Tregs employ PD-L1 to suppress the immune response in the BM to protect stem cell niches (15–18). In addition, STING activation in primary tumors has been shown to increase Treg activity and proliferation (59). Flow cytometry measurements of CD25^+^ FOXP3^+^ Tregs as a percentage of total CD4^+^ T cells demonstrate a significantly higher amount in treated BM versus control at day 14 (Fig. 4C). The combination of the naturally high concentration of Tregs in the BM and STING-mediated increase in Treg activity and proliferation are likely contributing factors to the therapy resistance at day 14.

## Discussion

The majority (>70%) of patients with metastatic breast cancer develop metastatic bone lesions with recurrence of their disease (60–62). The BM is a therapy-resistant microenvironment (9, 23, 63, 64), and consequently, breast tumor establishment in this niche has been challenging to treat and considered incurable (39, 65). At steady state, the BM assumes an immunosuppressive milieu to protect resident HSCs from self-recognition and to regulate their differentiation into immune progenitor cells (15–18). However, tumor cells capitalize on the relatively weak immune surveillance program of the BM TME. Metastatic solid tumors evoke multiple mechanisms to further foster an immunosuppressive microenvironment to evade immune detection, which has prompted the recent exploration of cGAMP and other CDN STING agonists as therapeutics to stimulate antitumor immunity. However, due to their poor pharmacologic properties, CDNs must be administered intratumorally to exert therapeutic effects, creating a significant technical barrier that has, to date, precluded evaluating their effect on the BM or the bone/TME. To address this limitation, we have utilized a recently described nanotechnology platform (i.e., STING-NPs) to systemically deliver 2′3′-cGAMP, the endogenous ligand for STING, allowing effects of STING activation on the BM and bone tumor growth to be examined (36, 37). Our findings demonstrate that systemic delivery of STING agonists can reduce tumor burden in an intratibial model of metastatic mammary carcinoma through reprogramming the immunosuppressive landscape of the BM.

Intravenous administration of 10 μg doses of cGAMP encapsulated in STING-NPs preferentially accumulated in the diseased hindlimb and reduced tumor burden at day 7 and decreased bone destruction at days 7 and 14 (Fig. 1C–H, 2A–G). STING activation has been shown to inhibit osteoclast differentiation *in vitro* through IFNβ signaling, which increased markedly in BM with treatment duration (Fig. 3A; ref. 66). However, comparable numbers of osteoclasts were observed at the healthy BM/bone interface for both STING-NP–treated and vehicle-treated mice at day 7 (Supplementary Fig. S5B), which suggests that the bone-protective effects of STING-NPs result from the initial reduction in tumor burden. The life span of osteoclasts is on the order of weeks, which can be extended when osteoclasts fuse with progenitor cells (67–69). Therefore, studying the effects of STING therapy on osteoclast differentiation and activity *in vivo* will require longer durations of treatment. These measurements could not be repeated for day 14 because the vehicle-treated control had minimal trabecular and cortical bone remaining. Investigating how systemic delivery of STING agonists affects bone turnover in nondiseased BM is an important future investigation as STING agonists are actively being explored clinical trials for treating primary lesions.

We postulate that the failure of the tumor to respond to treatment at day 14 (Fig. 1E and F) is a result of multiple resistance mechanisms that serve to dampen chronic inflammation in the BM in an attempt to return the tissue to *restitutio ad integrum*. At early timepoints, the cytokine signature of treated mice is indicative of STING activation with high IFNβ and CXCL10 signaling. This inflammatory program was accompanied by a significantly higher expression of early activation marker CD69 on both CD4^+^ and CD8^+^ T cells in the diseased BM of treated compared with PBS on day 7 (Fig. 4B). However, this cytokine signaling is diminished at day 14 due to anti-inflammatory mediators. For example, IL10 negatively regulates STING activity in part through triggering the degradation of IFNα/β receptor (70). IL10 was significantly lower at day 7 in treated BM compared with untreated but not at day 14. This increase in IL10 coincides with decreasing proinflammatory cytokines, creating a more favorable environment for tumor growth. We also hypothesize that IL2 plays a prominent role in the attenuation of the STING-NPs’ effects. While IL2 has been used to therapeutically stimulate the immune system and has recently been shown to synergize with STING agonists and checkpoint inhibitors in mouse models of primary mammary carcinoma (71), the role of IL2 as a proinflammatory or anti-inflammatory mediator is determined by the immunologic composition of the tissue of interest (72). In the BM where the concentration of Tregs is high, IL2 expression is likely to induce peripheral proliferation of this immunosuppressive population. This hypothesis was evaluated by performing flow cytometry analysis of CD4^+^ CD25^+^ FOXP3^+^ Tregs in diseased BM (Fig. 4C). On day 7 of the STING-NP treatment, there was no significant difference between number of Tregs out of total CD4^+^ cells in treated versus untreated cohorts. However, by day 14, a significant 3-fold increase in Tregs was observed in treated mice compared with untreated at the same timepoint. Furthermore, at the same timepoint, the expression of PD-L1 (i.e., CD279) increased by several fold on both CD4^+^ and CD8^+^ T cells in treated cohort. Tregs and the PD-L1/PD-1 pathway mediate immune suppression, likely contributing to reduced tumor outcomes on day 14 (Fig. 4B). The effects of STING-NP treatment on BM Treg populations were also noted in healthy mice (Supplementary Fig. S8). STING-NP treatment initially lowered the amount of Tregs in disease-free marrow, but the BM returned to its immune homeostasis by day 14. Taken together, these findings indicate that the BM of STING-NP–treated mice was inflamed by cGAMP delivery at early timepoints, but the BM TME upregulated inhibitory pathways to dampen the effect of STING-NPs.

CD11b^+^ myeloid cells compose approximately 75% of all BM mononuclear cells and 44% of all BM cells (73). Small changes in the heterogeneous subsets of this population can reshape how the BM responds to infection and disease. Prior to metastasis, it has been shown that signaling from primary 4T1 tumors increases granulocytes and cMOs and decreases DCs in the BM to establish a premetastatic niche at distant sites and prevent immune detection during metastasis (74). In our study, the number of granulocytes in the BM of STING-treated mice was significantly lower compared with untreated BM for all timepoints measured and CD11c^+^ cells were significantly higher on days 3 and 7. These changes are consistent with a myeloid composition that would lead to the improved tumor outcomes observed on day 7. By day 14 when the tumor had occupied most of the medullary volume of treated BM, the cMO concentration was 3-fold higher than the untreated. Monocytes and monocyte-derived suppressor cells have long been considered nefarious actors in the metastatic cascade of breast tumors (75, 76). Their accumulation is often a result of prolonged inflammatory states common with cancerous microenvironments. It is possible that long durations of STING activation trigger a response by these suppressor populations but further investigation is needed. Treating non–tumor-bearing mice with STING-NPs resulted in a precipitous 3.5-fold drop in CD11b^+^ staining in healthy BM after 7 days of treatment (Supplementary Fig. S6). While this effect was only transient, recovering to basal levels on day 14, STING NPs could be investigated as a prophylactic therapy for disrupting premetastatic niches after surgical resection of high-risk primary tumors. A short regimen of STING NPs could prevent surgery-induced cancer cell dissemination from seeding in healthy tissues (77).

This study was constrained by the limited number of mouse models of bone metastases. The 4T1-592 bone clone model is well known to establish in BM within a few days, which was necessary to avoid long-term treatment with STING-NPs. However, the findings from this study suggest that combination of STING-NPs with therapies that inhibit Tregs and immunosuppressive myeloid cells, such as ramucirumab and ipilimumab (78, 79), could expand the therapeutic window of STING-NPs past 7 days. In addition, it is also common to combine STING agonists with immune checkpoint blockade antibodies targeting the PD-1/PD-L1 pathway, which has been shown to have increased expression in our model. For slower growing bone metastases, pulsing STING-NP treatment could reprogram the immune response in the BM over multiple treatment cycles. Furthermore, conjugating bone affinities, such as bisphosphonates, to the outside of the STING-NPs could enable a lower dose with sustained and controlled release of cGAMP to the BM with reduced toxicity. This bone-targeted approach could also be used for mobilizing the BM to produce effector cells for primary tumor treatment. Such studies will be the focus of future investigations building from the current work.

## Supplementary Material

Figure S1 Supplementary Figure 1: Gating scheme for myeloid flow panel.

Figure S2 Supplementary Figure 2: Gating scheme for T cell activation flow panel.

Figure S3 Supplementary Figure 3: Gating scheme for Regulatory T Cell flow panel.

Figure S4 Supplementary Figure 4: Effects of STING-NP on mouse weights.

Figure S5 Supplementary Figure 5: TRAP+ Osteoclasts in treated versus untreated tibiae.

Figure S6 Supplementary Figure 6: Concentration of CD11B+ cells in healthy STING NP-treated BM over time.

Figure S7 Supplementary Figure 7: Systemic effects of STING-NP on circulating myeloid cells.

Figure S8 Supplementary Figure 8: Concentration of FOXP3+ cells in healthy STING NP-treated BM over time.
