# Supplementary figures and images for: Nanoparticle STING Agonist Reprograms the Bone Marrow to an Antitumor Phenotype and Protects Against Bone Destruction
Source: Cancer Res Commun. 2023 Feb 8;3(2):223–34. doi: 10.1158/2767-9764.CRC-22-0180 (PMC10035525; doi:10.1158/2767-9764.CRC-22-0180)

S1

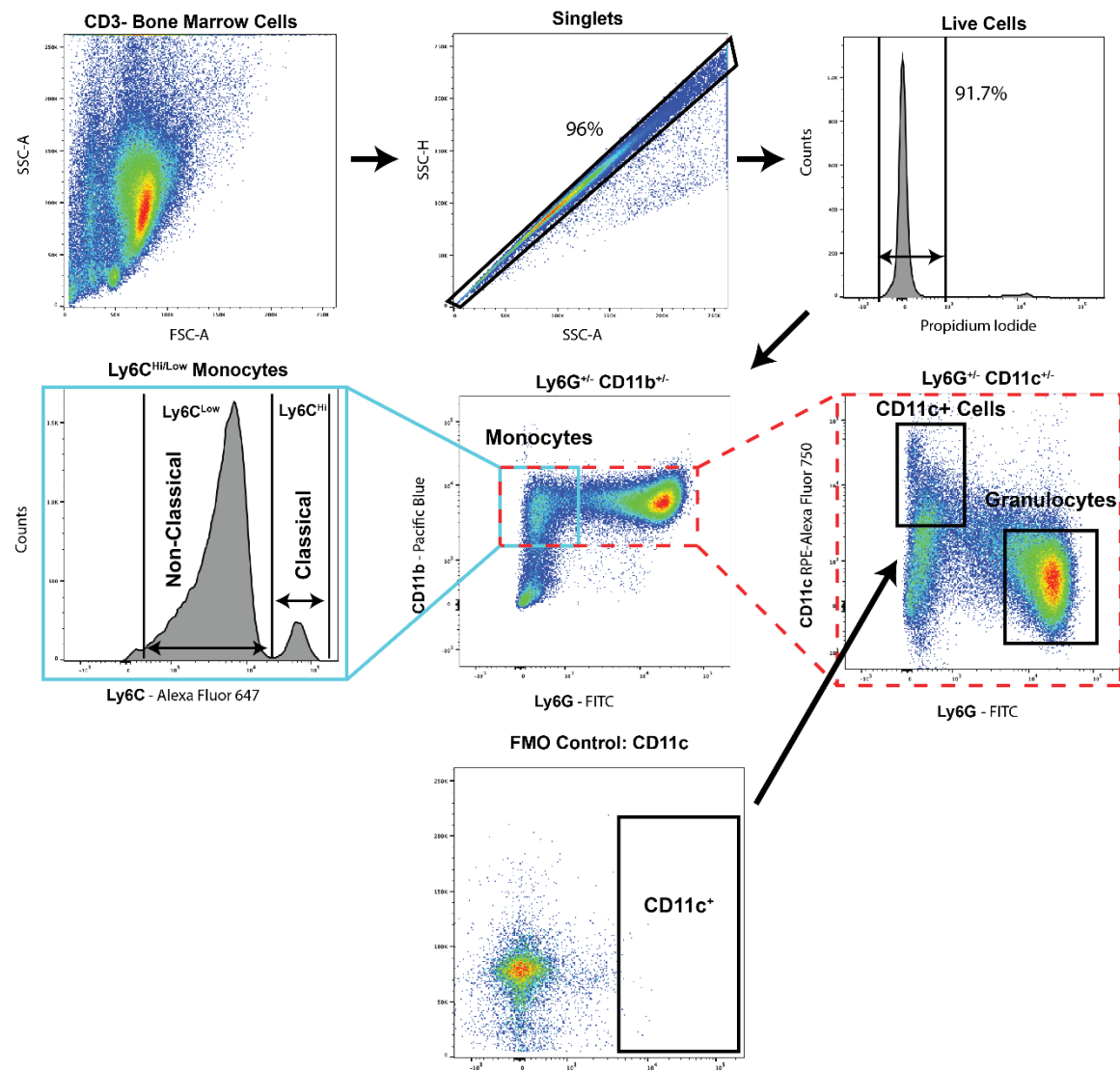

Supplementary Figure 1: Gating scheme for myeloid flow panel.

Supplement: Figure S1 — Supplementary Figure 1: Gating scheme for myeloid flow panel. [file crc-22-0180-s01.pdf]

S2

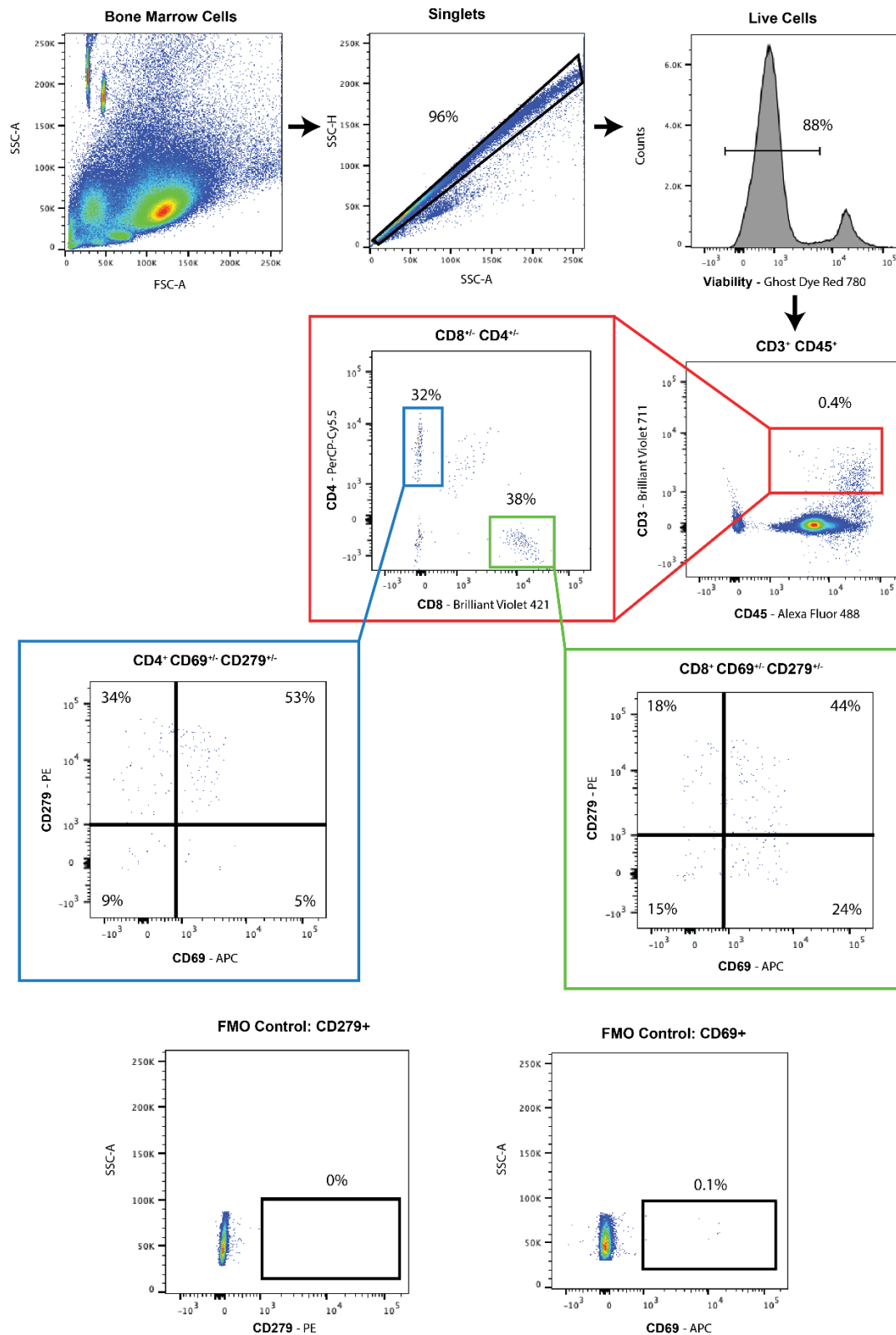

Supplementary Figure 2: Gating scheme for T cell activation flow panel.

Supplement: Figure S2 — Supplementary Figure 2: Gating scheme for T cell activation flow panel. [file crc-22-0180-s02.pdf]

S3

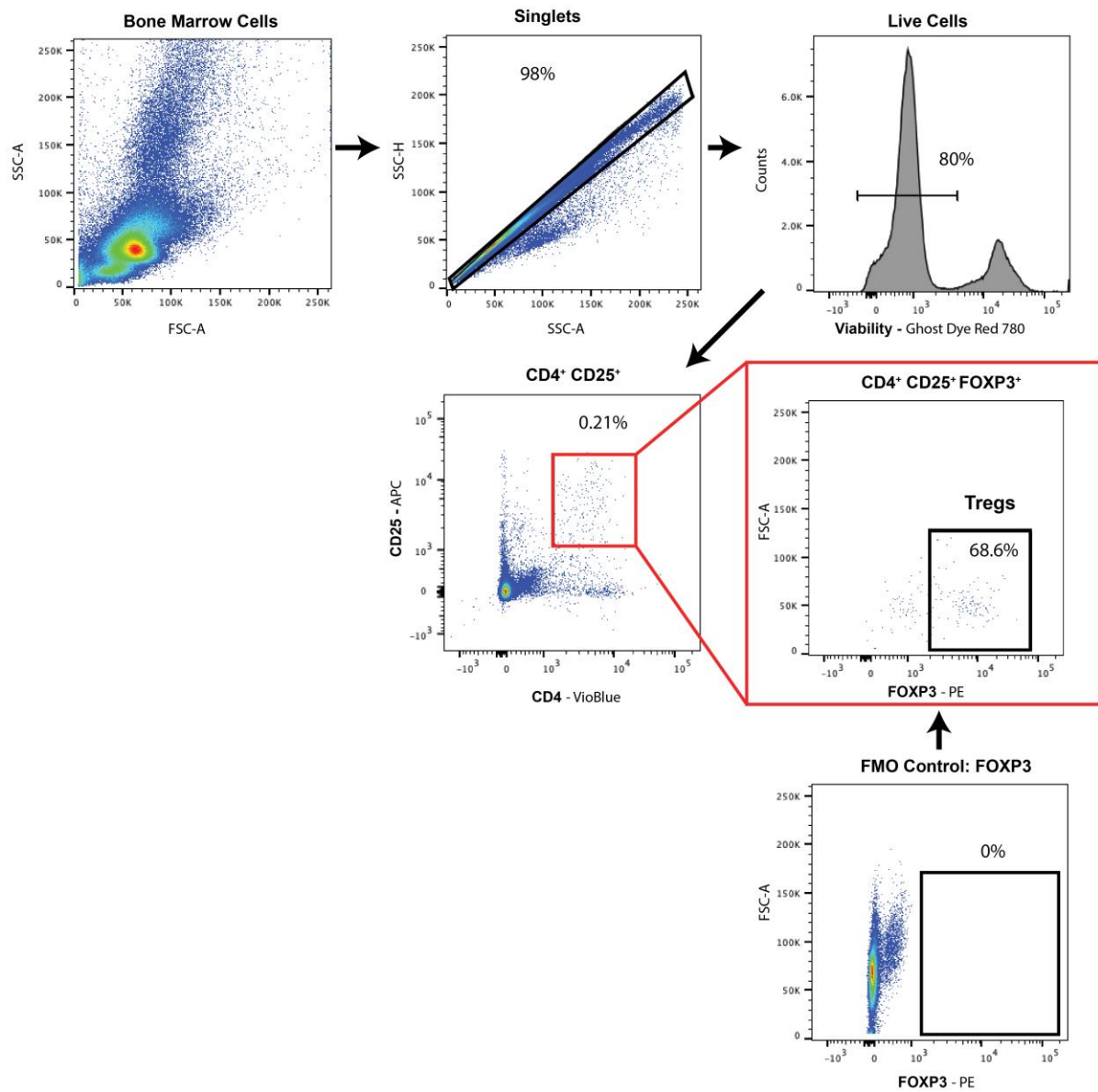

**Supplementary Figure 3: Gating scheme for Regulatory T Cell flow panel.**

Supplement: Figure S3 — Supplementary Figure 3: Gating scheme for Regulatory T Cell flow panel. [file crc-22-0180-s03.pdf]
