# Supplementary material for: Nanoparticle STING Agonist Reprograms the Bone Marrow to an Antitumor Phenotype and Protects Against Bone Destruction
Source: Cancer Res Commun. 2023 Feb 8;3(2):223–34. doi: 10.1158/2767-9764.CRC-22-0180 (PMC10035525; doi:10.1158/2767-9764.CRC-22-0180)
Supplement: Figure S4 — Supplementary Figure 4: Effects of STING-NP on mouse weights. [file crc-22-0180-s04.pdf]

S4

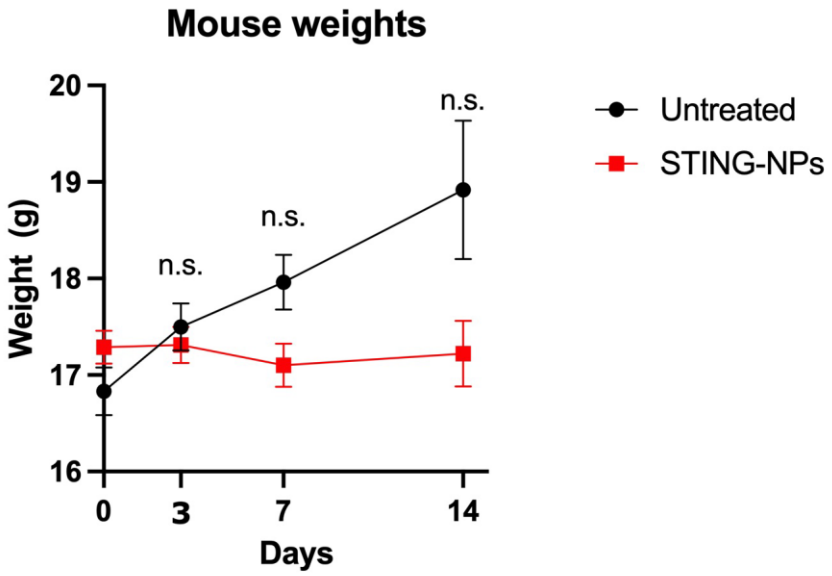


**Supplementary Figure 4: Effect of STING-NPs on mouse weight.** Mice (n=8) were weighed on days 0, 3, 7, and 14 of the experiment. Control mice weighed 16.8 g ± 1.0 g on day 0 and 18.9 g ± 1.6 g on day 14. STING-NP treated mice weighed 17.3 g ± 0.6 g on day 0 and 17.2 g ± 0.8 g on day 14. Multiple t-tests with Bonferroni correction. *: $p < 0.05$, **: $p < 0.01$, ***: $p < 0.001$. Error bars: s.e.m.
