# Supplementary material for: Nanoparticle STING Agonist Reprograms the Bone Marrow to an Antitumor Phenotype and Protects Against Bone Destruction
Source: Cancer Res Commun. 2023 Feb 8;3(2):223–34. doi: 10.1158/2767-9764.CRC-22-0180 (PMC10035525; doi:10.1158/2767-9764.CRC-22-0180)
Supplement: Figure S5 — Supplementary Figure 5: TRAP+ Osteoclasts in treated versus untreated tibiae. [file crc-22-0180-s05.pdf]

S5

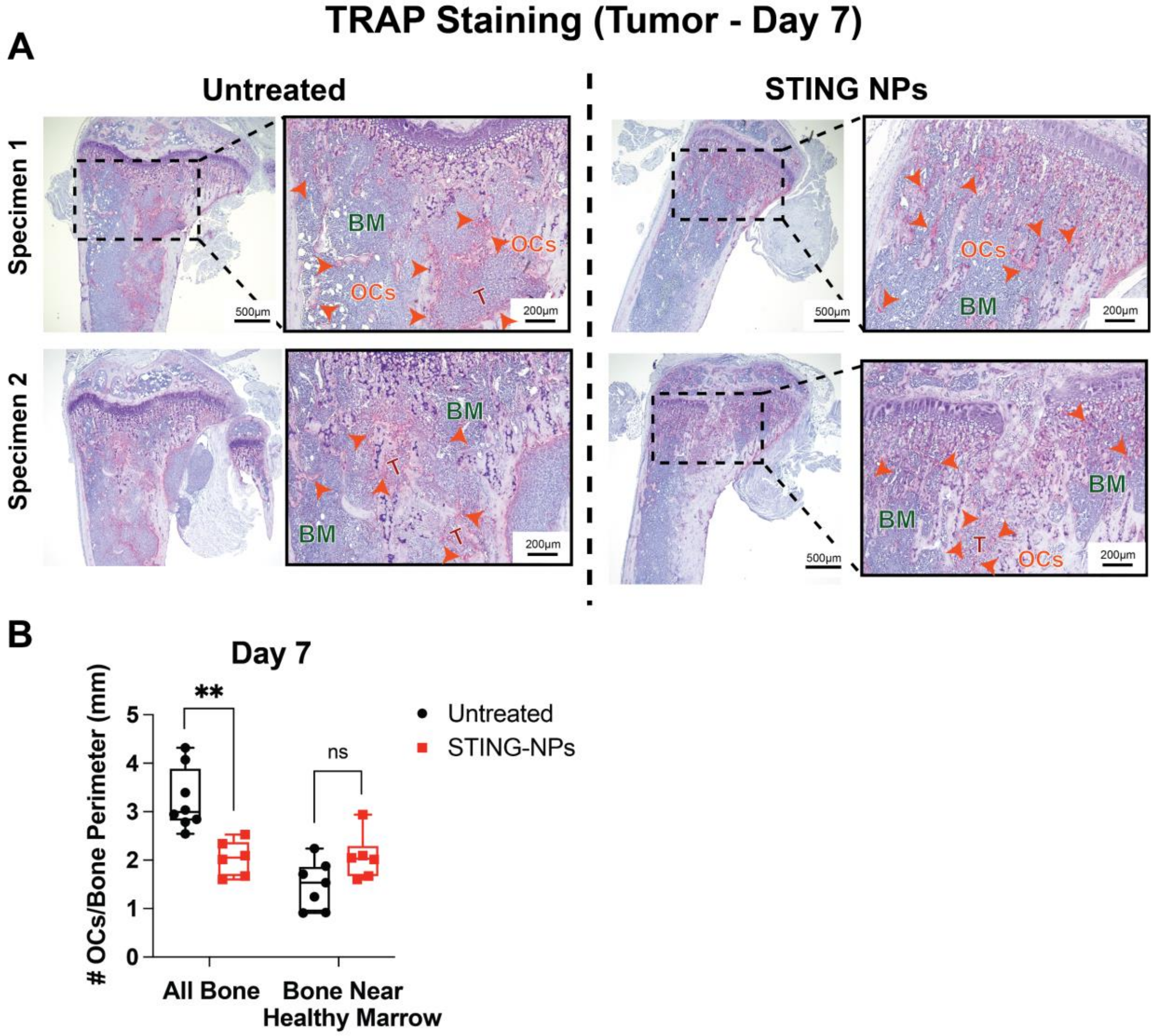

Supplementary Figure 5: TRAP+ Osteoclasts in treated versus untreated tibiae. Tibiae sections from mice with 7 days of tumor ± treatment (n=7) were stained for Tatrate-Resistant Acid Phosphatase (TRAP). (A) Osteoclasts were identified as TRAP-positive cells with 3 or more nuclei juxtaposed to trabecular bone. (B) Osteoclasts were counted and normalized to the entire trabecular bone perimeter using ImageJ. A subset of Osteoclasts located next to healthy bone marrow was also quantified.
