# Supplementary material for: Nanoparticle STING Agonist Reprograms the Bone Marrow to an Antitumor Phenotype and Protects Against Bone Destruction
Source: Cancer Res Commun. 2023 Feb 8;3(2):223–34. doi: 10.1158/2767-9764.CRC-22-0180 (PMC10035525; doi:10.1158/2767-9764.CRC-22-0180)
Supplement: Figure S6 — Supplementary Figure 6: Concentration of CD11B+ cells in healthy STING NP-treated BM over time. [file crc-22-0180-s06.pdf]

S6

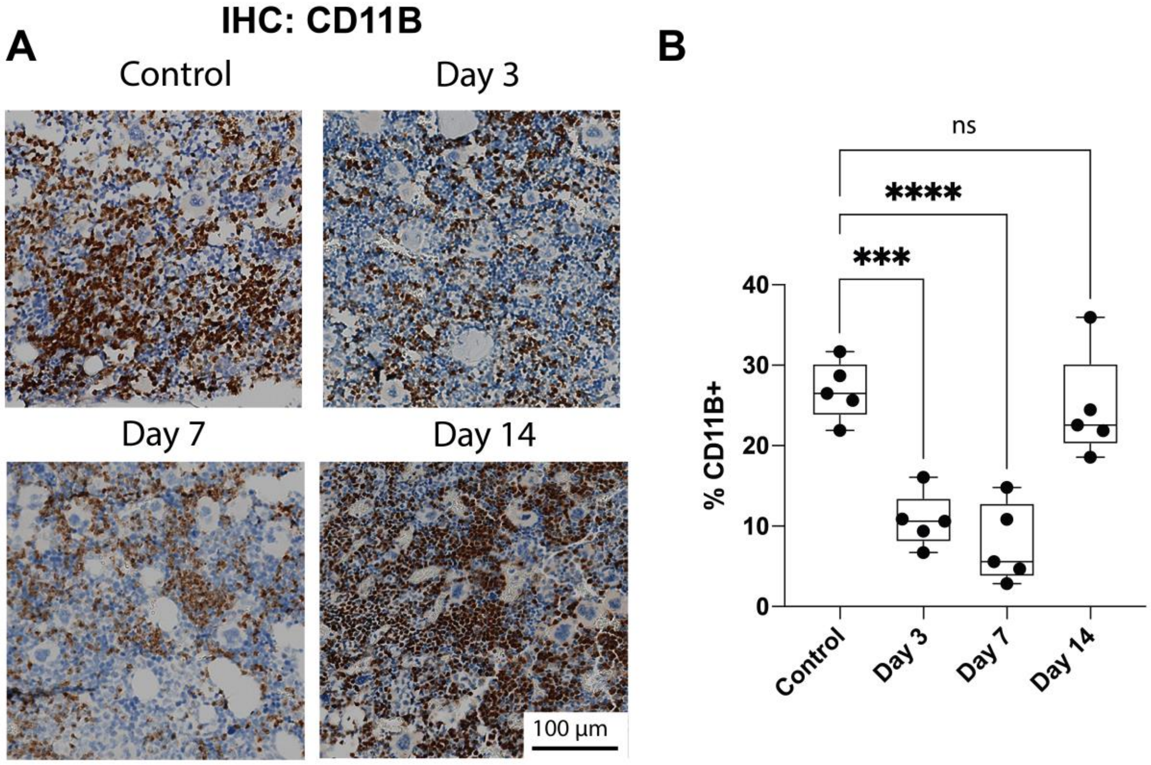

Supplementary Figure 6: **Concentration of CD11B+ cells in healthy STING NP-treated BM over time.** IHC staining of CD11B+ cells in healthy BM of untreated mice (control) and from mice that received the standard STING-NP treatment regimen (n=6). Mice were not tumor-bearing. One-way ANOVA with Holm-Šídák's multiple-comparisons test. *: $p < 0.05$, **: $p < 0.01$, ***: $p < 0.001$, ****: $p < 0.0001$.
