# Supplementary material for: Nanoparticle STING Agonist Reprograms the Bone Marrow to an Antitumor Phenotype and Protects Against Bone Destruction
Source: Cancer Res Commun. 2023 Feb 8;3(2):223–34. doi: 10.1158/2767-9764.CRC-22-0180 (PMC10035525; doi:10.1158/2767-9764.CRC-22-0180)
Supplement: Figure S7 — Supplementary Figure 7: Systemic effects of STING-NP on circulating myeloid cells. [file crc-22-0180-s07.pdf]

S7

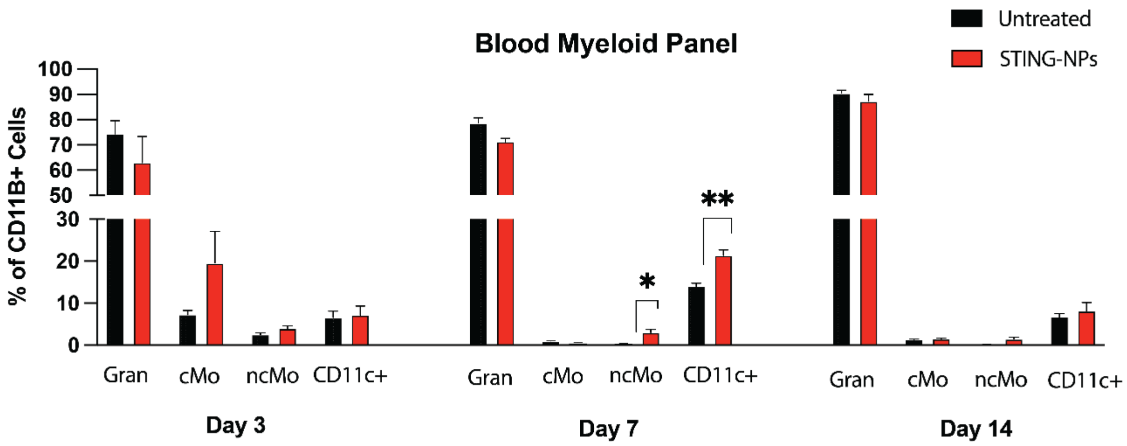

Supplementary Figure 7: Systemic effects of STING-NP on circulating myeloid cells. Flow cytometry analysis of blood from tumor-bearing hindlimbs of STING-NP treated and untreated mice at days 3, 7, and 14 (n=6). Granulocytes (Gran:CD11b+ CD11c- Ly6G+), Classical Monocytes (cMo: CD11b+ Ly6Chi CD11c- Ly6G-), Non-Classical Monocytes (ncMo: CD11b+ Ly6Clo CD11c- Ly6G-), and CD11c+ Cells (CD11c+ CD11b+ Ly6C+/- Ly6G+/-) were quantified as a percentage of CD11B+ cells. Multiple t-tests with Bonferroni correction. *: $p < 0.05$, **: $p < 0.01$, ***: $p < 0.001$ Error bars: s.e.m.
