# Supplementary material for: Nanoparticle STING Agonist Reprograms the Bone Marrow to an Antitumor Phenotype and Protects Against Bone Destruction
Source: Cancer Res Commun. 2023 Feb 8;3(2):223–34. doi: 10.1158/2767-9764.CRC-22-0180 (PMC10035525; doi:10.1158/2767-9764.CRC-22-0180)
Supplement: Figure S8 — Supplementary Figure 8: Concentration of FOXP3+ cells in healthy STING NP-treated BM over time. [file crc-22-0180-s08.pdf]

S8

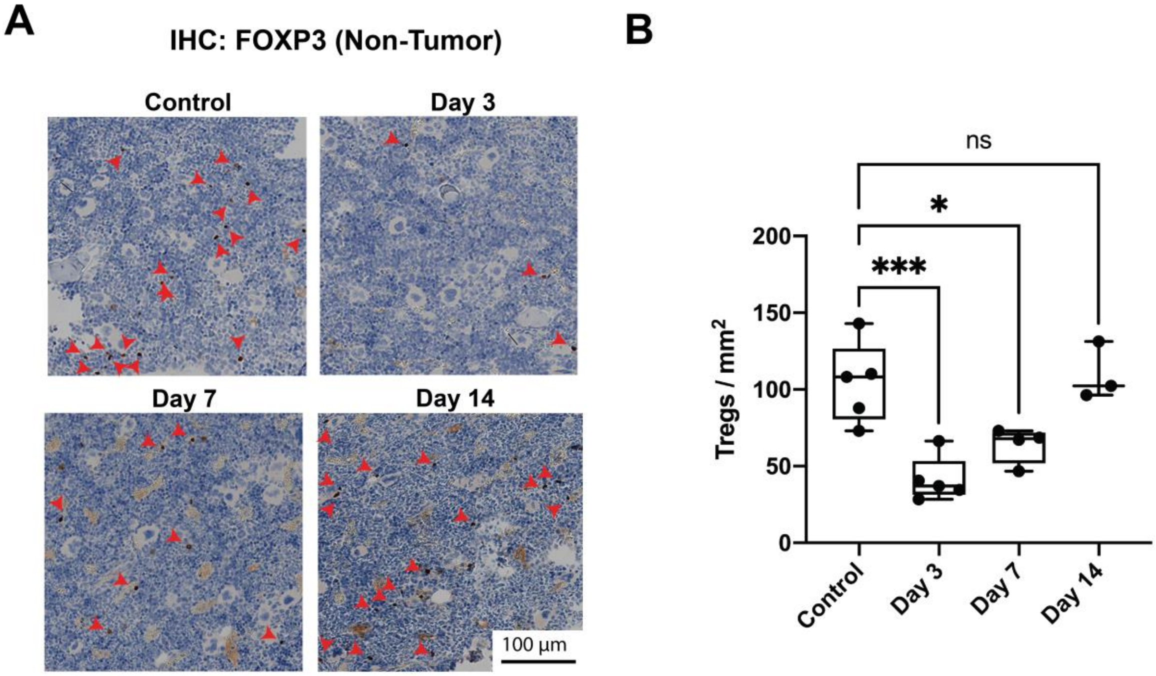

Supplementary Figure 8: Concentration of FOXP3+ cells in healthy STING NP-treated BM over time. (A) IHC staining of FOXP3+ Tregs in healthy BM from untreated mice (control) and from mice that received the standard treatment regimen. Mice were not tumor-bearing. (B) FOXP3+ cells were counted individually and normalized to total marrow area. One-way ANOVA with Holm-Šídák's multiple-comparisons test. *: $p < 0.05$, **: $p < 0.01$, ***: $p < 0.001$
